# Construction of a Novel Lipase Catalytic System Based on Hybrid Membranes with Interwoven Electrospun Polyacrylic Acid and Polyvinyl Pyrrolidone Gel Fibers

**DOI:** 10.3390/gels8120812

**Published:** 2022-12-10

**Authors:** Ziheng Wang, Shumiao Lin, Qianqian Zhang, Jinlong Li, Sheng Yin

**Affiliations:** Beijing Engineering and Technology Research Center of Food Additives, Beijing Technology and Business University, Beijing 100048, China

**Keywords:** lipase, interfacial catalysis, hybrid gel fiber membrane, double-needle electrospinning

## Abstract

Efficient lipase catalysis requires sufficient oil–water interface engineered through structural design. Inspired by the architectural features of fabrics, a novel lipase-membrane catalytic system with interwoven polyacrylic acid (PAA) gel fibers and polyvinyl pyrrolidone (PVP) gel fibers was developed in this study by using double-needle electrospinning and gelation. It has been demonstrated that PAA/PVP hybrid gel fiber membranes (HGFMs) have a high swelling capacity for both water and oil phases, which created numerous discontinuous oil–water contact surface units in limited space of HGFMs, consequently forming effective interfacial catalytic systems. Volume competition between the water and oil phases suggests that balancing the proportions of these phases is very important for effective construction of oil–water interfaces and conditioning catalysis. Regulation of multiple factors of PAA/PVP HGFMs resulted in a catalytic efficiency of up to 2.1 times that of a macroscopic “oil-up/water-down” system (room temperature, pH = 7), and 2.9 times when three membranes are superimposed, as well as excellent pH and temperature stability. HGFMs were stacked to build a high-performing catalytic performance reactor. We expect that this study will be a beneficial exploration for expanding the lipase catalytic system.

## 1. Introduction

Lipase (EC 3.1.1.3), also known as triglyceride hydrolase, is a biocatalyst that has been widely used in food processing, medicine and hygiene, chemical synthesis, environmental protection, energy development, biosensors and many other fields [1,2,3,4,5,6,7] because of its applicability to many types of reactions, such as hydrolysis, esterification, transesterification, and ammonolysis, with chemoselectivity and regioselectivity [1,4,8,9,10,11].

Lipase is a member of the interfacial enzyme family [9] and has a catalytic active center covered by one or more amphiphilic α-helical loops (also known as “lid” structures) [12,13]. This unique molecular structure endows lipase with particular interfacial catalytic characteristics. Specifically, the “lid” conformation changes from “closed” to “open” when lipase contacts a hydrophilic–hydrophobic interface, which promotes specific binding between the substrate and the active center of lipase [14,15,16,17,18]. 

Research on lipase catalysis has always been focused on interfacial catalysis, which requires that the formation interfaces between the hydrophobic phase (containing the substrate) and the hydrophilic phase (containing lipases). Therefore, a sufficiently large and effective catalytic interfacial area is very important to ensure good catalytic performance. Many studies have been performed on using emulsions (microemulsions, nanoemulsions, Pickering emulsions, etc.) to create sufficient oil–water catalytic interfaces for lipase catalysis [19,20,21,22]. However, there are shortcomings associated with using traditional emulsions for this purpose. (1) The microemulsion forms a very complex interfacial system, it is difficult to select a suitable surfactant, and the enzyme loading is insufficient. (2) Oil-in-water (O/W) and water-in-oil (O/W) emulsions have poor stability and are easily decomposed by environmental factors [12,23]. (3) The recycling rate of lipase is low, and the product is not easy to purify [24,25]. By comparison, immobilizing lipase with materials of different dimensions and structures offers considerable advantages for industrial production. Most immobilization strategies rely on the structural characteristics of different materials to achieve specific binding or nonspecific adsorption between materials and lipases, thereby improving the activity and stability of lipases, however little attention has been paid to regulating lipase performance from an oil-water interface perspective [26,27,28]. 

**Figure 1:**
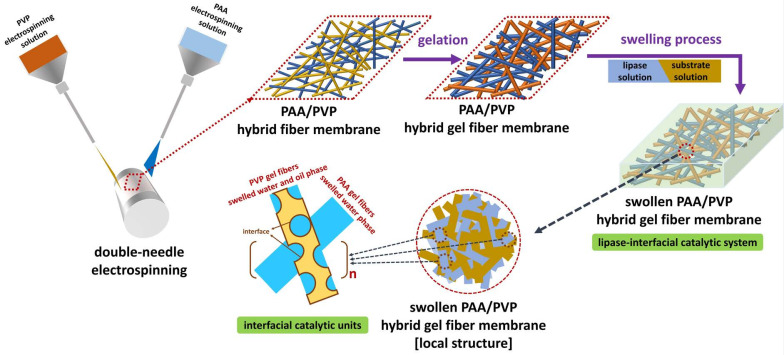
Illustration of the two-step preparation strategy of PAA/PVP hybrid gel fiber membranes based on double-needle electrospinning and gelation and the construction of an interfacial catalytic system for lipase.

In the current study, inspired from the typical interwoven structural features of fabrics, a novel lipase catalytic system was developed: a simple two-step strategy (Figure 1) that combined double-needle electrospinning and gelation was used to interweave polyacrylic acid (PAA) gel fibers and polyvinyl pyrrolidone (PVP) gel fibers to form electrospun PAA/PVP hybrid gel fiber membranes (HGFMs). First, PAA/PVP hybrid fiber membranes (HFMs) were fabricated by double-needle electrospinning, and hybrid gel fiber membranes (HGFMs) were then formed by using thermal crosslinking to transform PAA fibers into ordered PAA gel fibers and using UV irradiation crosslinking to transform PVP fibers into PVP gel fibers. As we imagined, PAA gel fibers had excellent water phase swelling performance, while PVP gel fibers has good oil phase wettability and a certain degree of water phase swelling capacity. Consequently, in the interior space of the HGFMs, lipases could be entrapped in hydrogel networks of the PAA and PVP gel fibers during water phase swelling, and substrate dissolved in dodecane could be entrapped in the uniquely lipophilic PVP gel fibers (containing lipophilic methylene) during the swelling of hydrophobic phase. Within the local structure of PAA/PVP HGFMs, a tiny oil-water interface exists between the hydrophobic region of PVP gel fibers and the hydrophilic region of PAA gel fibers, as well as between the hydrophobic and hydrophilic regions of PVP gel fibers. Although each tiny interface has insufficient catalytic efficiency, “micro-macro, finite-infinite” logic leads to the expectation that the whole PAA/PVP HGFM has a considerable total catalytic efficiency. The hybrid system of dual gel fibers also offers clear advantages for enzyme loading, and the porous structure of the fibers facilitates product purification [3,29]. Since it combined the advantages of both gel technology and electrospinning technology, we expect that this study will be a beneficial exploration for expanding the lipase catalytic system.

## 2. Results and Discussion

### 2.1. Structures of PAA/PVP HFMs and PAA/PVP HGFMs

**Figure 2:**
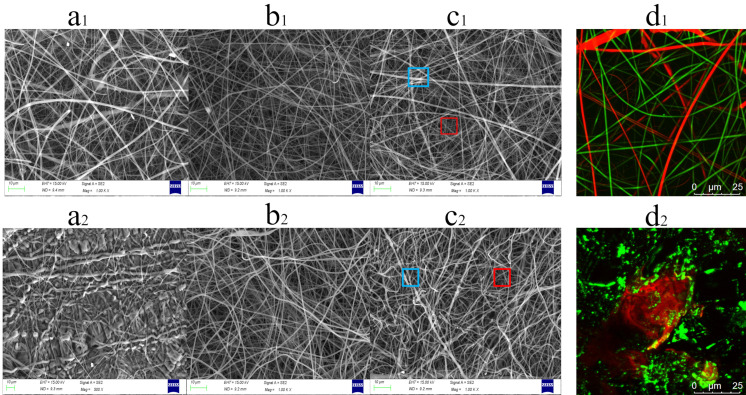
SEM images of PAA fiber membranes (**a_1_**), PVP fiber membranes (**b_1_**), PAA/PVP HFMs (**c_1_**), PAA gel fiber membranes (**a_2_**), PVP gel fiber membranes (**b_2_**), PAA/PVP HGFMs (**c_2_**), CLSM images of PAA/PVP HFMs (**d_1_**) and PAA/PVP HGFMs (**d_2_**). The blue box in c_1_ and c_2_ indicates PAA fibers and PAA gel fibers, respectively, and the red box in c_1_ and c_2_ indicates PVP fibers and PVP gel fibers, respectively. The membranes were prepared using a thermal crosslinking time of 45 min, and a UV-crosslinking time of 15 min. An electrospinning speed ratio of PAA/PVP of 5/8 was used to obtain the membranes shown in (**a**–**c**).

In the current study, a simple two-step strategy combining double-needle electrospinning and gelation (Figure 1) was adopted to fabricate PAA/PVP HGFMs. Figure 2a_1_,b_1_ show that the diameter of PAA electrospun fibers was larger than that of PVP electrospun fibers, which confirmed the fabrication of hybrid fiber membranes with interwoven structures of PAA electrospun fibers and PVP electrospun fibers (Figure 2c_1_). FTIR analysis (Supplementary Figure S1) indicated that thermal and UV crosslinking successfully converted PAA/PVP HFMs to PAA/PVP HGFMs. Two-step crosslinking to convert PAA fibers to PAA gel fibers resulted in a prominent change in the PAA fiber morphology, whereas the conversion of PVP fibers to PVP gel fibers did not result in a significant change in the PVP fiber morphology. The difference in the diameters of the PAA gel fibers and PVP gel fibers was used to easily confirm the composition of the HGFMs (Figure 2c_2_).

When fluorescent dyes were premixed into the electrospinning solutions, the CLSM images (Figure 2d_1_,d_2_) confirmed the components and structural features of the HFMs and HGFMs, as well as the dual-swelling capacity of PAA/PVP HGFMs (a detailed discussion is presented in Section 2.2).

### 2.2. Wettability and Swelling Properties of PAA/PVP HGFMs

**Figure 3:**
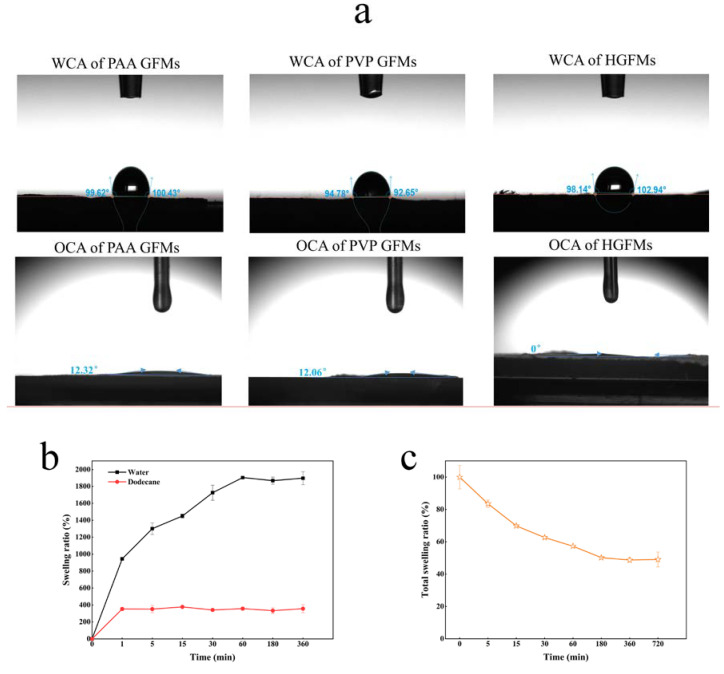
Wettability and swelling properties of different fiber membranes (**a**); WCA and oil contact angle (OCA) of different fiber membranes (**b**); swelling curve of HGFMs as a function of time (**c**); swelling curve of HGFMs as a function of time obtained by using a procedure of saturated swelling with water followed by infiltration with dodecane. The following parameters were used to prepare the HGFMs corresponding to a-c: an electrospinning speed ratio of PAA/PVP of 5/8, an electrospinning time of 8 h, a thermal crosslinking time of 45 min, and a UV crosslinking time of 15 min.

The prepared PAA GFMs and PVP GFMs, as the two control groups, exhibited different affinities for the water phase. Figure 3a shows that the water contact angle of PVP GFMs was slightly lower than that of PAA GFMs, which may be related to the fact that the PVP fiber membrane did not shrink significantly after crosslinking and the presence of numerous voids in the fiber membrane (see Figure 2b_2_). Note that the slight hydrophobicity of the prepared PAA/PVP HGFMs does not imply that the PAA/PVP HGFMs could not sufficiently wet the water phase, because only a sufficient time for water absorption to swell the membrane was required (see Figure 3b). The PAA/PVP HGFMs had an oil contact angle of only 0°, indicating that the oil phase (dodecane) effectively infiltrated into the HGFMs (1 min was required for saturated swelling, see Figure 3b). The infiltration results for both the water and oil phases also indicated that although the prepared PAA/PVP HGFMs had a swelling capacity for both the water and oil phase (dodecane) (which is the premise for constructing oil-water interfaces in PAA/PVP HGFMs), the swelling behavior of the water phase was very different from that of the oil phase (Figure 3b). An interesting phenomenon was that when the PAA/PVP HGFMs were first swollen to saturation with water and then infiltrated with dodecane, the total swelling ratio decreased with increasing dodecane phase infiltration (Figure 3c), indicating that the total weight of the swollen PAA/PVP HGFMs decreased with increasing dodecane soaking time. We submit that dodecane infiltration into the space of the water phase in the PVP gel fibers was thus facilitated, resulting in the extrusion of the water phase.

**Figure 4:**
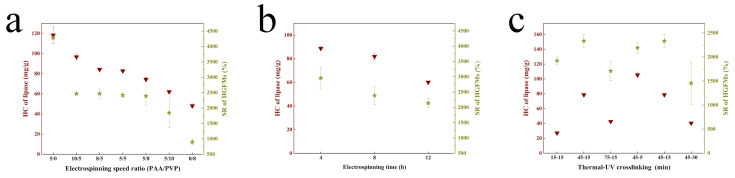
Lipase-holding capacities and swelling ratios of PAA/PVP HGFMs determined under different experimental conditions. (**a**) Different electrospinning speed ratios. (**b**) Different electrospinning times. (**c**) Different crosslinking times.

Generally consistent trends were observed for the effect of the PAA proportion (as controlled by the electrospinning speed ratio) on the swelling capacity and lipase-holding capacity of the HGFMs (Figure 4a). In general, the higher the PAA proportion, the higher the swelling capacity and lipase-holding capacity of the PAA/PVP HGFMs were. These consistent trends confirmed that lipases entered the PAA/PVP HGFMs during aqueous phase swelling.

The influence trend of the electrospinning time and the two-step crosslinking time on the water-phase swelling ratio was basically the same as that on the lipase-holding capacity of the PAA/PVP HGFMs (Figure 4b,c). The optimal times for maximizing the water-phase swelling ratio and lipase-holding capacity were 4h for electrospinning, 45 min for thermal crosslinking and 5 min for UV crosslinking. Note that with increasing electrospinning time, the swelling ability and lipase-holding capacity of the HGFMs trended downward, indicating that increasing the membrane thickness inhibited entry of the water phase and lipase.

### 2.3. Catalytic Efficiency of PAA/PVP HGFMs

**Figure 5:**
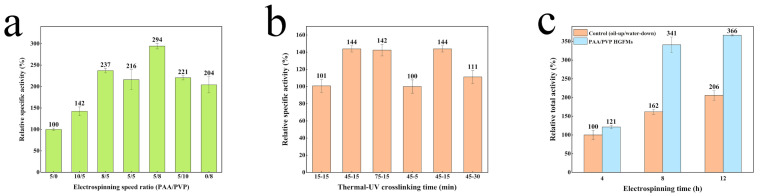
Influence of the component ratio (**a**), crosslinking time (**b**) and electrospinning time (**c**) on the catalytic efficiency of lipase-PAA/PVP HGFM systems. The results shown in (**a**,**c**) were obtained using a thermal crosslinking time of 45 min and a UV crosslinking time of 15 min. The results shown in (**a**,**b**) were obtained using an electrospinning time of 8 h. The interfacial area of the macroscopic “oil-up/water-down” system was set equal to the cross-sectional area of the HGFMs.

The preparation process of the PAA/PVP HGFMs had a significant effect on the catalytic activity for lipase. Figure 5a shows that the pure PAA GFMs exhibited the lowest catalytic activity because of the poor ability of the PAA gels to swell the oil phase (the SR of the oil phase ≈ 50%), resulting in almost no oil–water interfacial area. Hence, increasing the PVP proportion (by controlling the electrospinning speed ratio) in the PAA/PVP HGFMs resulted in a significant increase in the catalytic activity, which was highest at a 5:8 electrospinning ratio. Further increasing the PVP proportion caused the catalytic activity for lipase to decrease. It was noteworthy that the pure PVP GFMs (prepared at an electrospinning speed ratio = 0/8) also showed some degree of catalytic activity, which is consistent with the ability of pure PVP GFMs to swell both the water and oil phases (the SR of the oil phase ≈ 300%), whereby some intra-oil-water interfaces formed in the PVP gels. There were two contributions to the oil-water interfacial area in the PAA/PVP HGFMs: (1) the intra-oil-water interfaces in the PVP gel fibers and (2) the inter-oil-water interfaces between the PAA gel fibers and PVP gel fibers. Hence, the area of the oil–water interfaces can be regulated by changing the ratio of the PAA gel fibers to the PVP gel fibers.

The crosslinking reaction was a key step in converting fibers into gel fibers. Figure 5b shows that an insufficient time for thermal or UV crosslinking resulted in a substantial decrease in the relative enzyme activity of lipase. An insufficient crosslinking time resulted in some PAA or PVP fibers not being incorporated into the gels, causing the collapse and dissolution of the fiber structure during swelling. The consistent trend between Figure 4b and Figure 5b also indicated an internal correlation among the catalytic activity, lipase-holding capacity and swelling ratio. We hypothesized that increasing the thermal crosslinking time resulted in tighter entanglement of the PAA gel fibers (Supplementary Figure S2), which may have promoted the catalytic activity of the PAA/PVP HGFMs.

However, the trend in Figure 5c was not consistent with that shown in Figure 4c. The relative total activity of lipase in the PAA/PVP HGFMs only increased by 21% over that of the control group for membranes prepared using an electrospinning time of 4 h but increased significantly upon increasing the electrospinning time to 8 h. However, although the lipases load per unit area of HGFMs increased upon increasing the electrospinning time further to 12 h, the quantity of lipases in the HGFMs increased significantly. The overall enzyme activity was not improved significantly, which may have resulted from the complex effect of the increase in the thickness of the HGFMs. A sufficient reaction time is required to enable the substrate solution to enter the interior space of the HGFMs.

**Figure 6:**
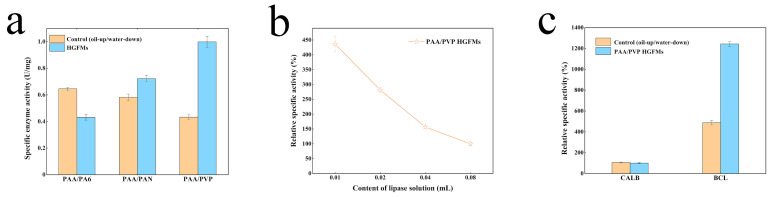
Influence of the component type (**a**), lipase content (**b**), and lipase type (**c**) on the catalytic efficiency of lipase-HGFM systems. The HGFMs were prepared using an electrospinning time of 8 h, a thermal crosslinking time of 45 min, and a UV crosslinking time of 15 min. A lipase concentration of 4 mg/mL was used to obtain the results shown in (**b**).

Supplementary Figure S3 shows significant morphological characteristic differences among the three hybrid membranes both before and after crosslinking, which means that the differences in catalytic activity was caused by the morphological differences among the three fibers (PA6/PAN/PVP). Figure 6a confirms the key role the PVP gel fibers played in promoting the catalytic efficiency of the HGFMs. The PA6 fibers had good hydrophilicity and acted as oleophobes in the PAA/PA6 HGFMs, which therefore exhibited the lowest catalytic efficiency, even below that of the control group (the oil-up/water-down system). The highly hydrophobic PAN fibers facilitated infiltration of the oil phase, endowing PAA/PAN HGFMs with the ability to both be swelled by the water phase and infiltrated by the oil phase, such that the catalytic efficiency of the PAA/PAN HGFMs was significantly promoted over that of the PAA/PA6 HGFMs and even higher than that of the control group (oil-up/water-down), while remaining lower than that of the PAA/PVP HGFMs; this result was obtained because the strongly hydrophobic PAN fibers severely inhibited the ability of PAA gel fibers to be swelled by the water phase. This result demonstrates that enhancement of the catalytic activity of lipase-HGFM systems require sufficient quantities of water and oil phases to form oil–water interfaces.

The distribution of the oil-water interfaces also affected the lipase activity. Figure 6b shows that with increasing lipase content (controlled by the volume of the lipase solution), the lipase activity decreased rapidly. Assuming a constant space vector for each prepared membrane, an increase in the proportion of the water phase (the lipase solution) implies a decrease in the volume occupied by the oil phase (the substrate solution). Comprehensively considering the results presented in Figure 3c, the volume competition between the water and oil phases suggests that balancing the proportions of these phases is very important for effective construction of oil-water interfaces.

Figure 6c shows that using lipases with and without a “lid” structure to construct lipase-HGFM systems resulted in completely different catalytic performances. The relative specific activity of CALB (without a “lid” structure) in the HGFMs was basically the same as that of the control group (the oil-up/water-down system), whereas BCL (with a “lid” structure) exhibited efficient catalytic performance. This finding validates our strategy for exploiting interfacial effects to design of hybrid membrane systems.

**Figure 7:**
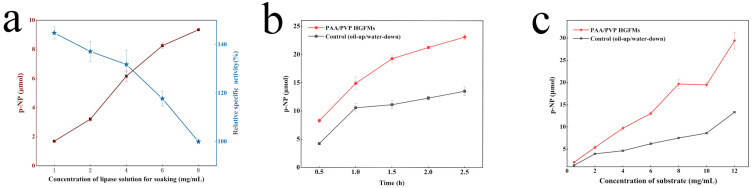
The catalytic capacity of the PAA/PVP HGFMs varied with different experimental factors: (**a**) the concentration of the lipase solution used to soak the HGFMs, (**b**) time during catalytic reaction process and (**c**) the substrate concentration used in the catalytic reaction process. All the HGFMs were prepared using an electrospinning speed ratio of PAA/PVP of 5/8. A thermal crosslinking time of 45 min and a UV crosslinking time of 15 min were used. An electrospinning time of 8 h was used.

Figure 7a shows that with increasing lipase concentration, an increasing number of lipase molecules assembled at the oil–water interface, thus improving the catalytic efficiency of the whole system. Note that the limited total oil–water interface area available for lipase restricted the available space for the assembly of lipase molecules. With increasing lipase concentration, the lipase molecules at the interface became increasingly crowded; however, even when no interfacial area remained to accommodate lipase molecules, the catalytic efficiency of the whole system continued to slowly increase. The decrease in the specific activity of lipase also shows that the loss of the effective oil–water interface area increased the number of lipase molecules that could not assemble at the interface to participate in the reaction.

The PAA/PVP HGFMs always maintained a higher catalytic rate than free lipase within 0−2.5 h (Figure 7b). With increasing catalysis time, the quantity of the product (*p*-NP) obtained by lipase catalysis in the HGFMs increased continuously; the catalytic reaction of lipase was suppressed after 1 h, indicating that the HGFMs continuously penetrated the oil phase to form a rich oil–water interface. Combining these results with those shown in Figure 3c and Figure 7c led to the conclusion that the infiltration of the oil phase during the catalytic HGFM reaction caused the water phase to separate out, transforming the gel fiber from a fully swollen to a semi-swollen state, which affected the increase in the quantity of subsequent HGFM products.

The catalytic efficiency of the free lipase and the HGFMs over time is shown in Figure 7c. With increasing substrate concentration, the catalytic performance of lipase in the HGFMs remained significantly higher than that of the control group, which indicated that the HGFMs resisted the inhibitory effect produced by increasing the substrate concentration and the excellent oil-wetting ability of the interior space (containing the substrate). This fluidity effectively prevented localized aggregation of substrates.

### 2.4. Application Performance of PAA/PVP HGFMs

**Figure 8:**
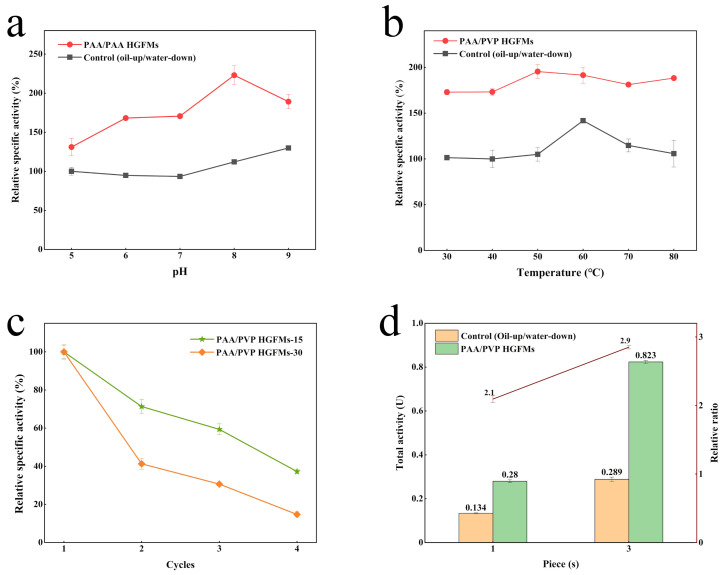
Application performance of the PAA/PVP HGFMs at different pH (**a**), temperatures (**b**), numbers of cycles for different reaction times (**c**) and different numbers of membrane pieces assembled in the reactors (**d**). All the HGFMs were prepared using an electrospinning speed ratio of PAA/PVP of 5/8, an electrospinning time of 8 h, a thermal crosslinking time of 45 min, and a UV crosslinking time of 15 min.

The pH and temperature played a pivotal role in the catalytic process for lipase. The effect of the pH and temperature on the hydrolysis efficiency of lipase is shown in Figure 8. The optimum pH of free lipase was 9 for the oil-up/water-down system and shifted to 8 for the HGFMs. The optimum temperature of free lipase for the oil-up/water-down system was 60 °C, whereas free lipase in the HGFMs maintained stable activity over a wide temperature range (30–80 °C). Regardless of the temperature and pH, the enzymatic activity of lipase was considerably higher in the HGFMs than in the oil-up/water-down system. The lipase in the HGFMs retained 37% and 15% of its activity after 4 cycles of catalytic reaction. With increasing reaction cycle time, the recycling rate of the lipase decreased. This result may have been obtained because of the long-term infiltration of the oil phase during the catalytic reaction, whereby the water phase in the HGFMs was precipitated (Figure 3c) and a part of the lipase easily leaked out with the water phase. With increasing reaction cycle time, the leakage of lipase gradually increased, resulting in a decrease in the lipase activity.

In the current study, the HGFMs were found to have a high catalytic efficiency and high loading capacity of lipase, as well as a high potential for constructing enzyme-membrane reactors. HGFMs are an interwoven membrane of two gel fibers and had a very low spatial occupancy rate, good foldability similar to that of fibers after swelling, and dual-holding ability for the water and oil phases. The superposition of the oil-up/water-down system in the vertical space (by increasing the quantity of the enzyme solution) was limited by the presence of the two-dimensional oil–water interface, and the lipase activity was not significantly improved. However, the HGFMs were not limited by the presence of the two-dimensional oil–water interface. There were a large number of oil–water interface units inside the HGFMs. For vertically stacked HGFMs, the internal oil–water interface still realized efficient lipase catalysis. In a space with a fixed size, the lipase catalytic efficiency of a single-sheet membrane of the HGFMs was 2.1 times that of the oil-up/water-down system, whereas the lipase catalytic efficiency of three-piece membranes was 2.9 times that of the oil-up/water-down system. The superposition of an infinite number of membranes could make lipase highly efficient in a limited space. The unit enzyme activity of the three membranes was found to be 96% that of one membrane, with almost no loss of enzyme activity. Therefore, the efficient catalytic performance of the monolithic membrane can be enhanced by using the HGFMs prepared in this study to assemble an enzyme-membrane reactor in a longitudinal space.

## 3. Conclusions

In summary, inspired by the typical interwoven structural features of fabrics, a series of PAA/PVP HGFMs for use as novel lipase catalytic systems were prepared by combining double-needle electrospinning and gelation. The study results confirmed that the high catalytic efficiency of PAA/PVP HGFMs resulted from the formation of numerous tiny oil-water interfaces with two contributions: (1) intra-oil-water interfaces in the PVP gel fibers and (2) inter-oil-water interfaces between the PAA gel fibers and PVP gel fibers. Hence, the area of the oil-water interfaces can be regulated by changing the ratio of the PAA gel fibers to the PVP gel fibers, and the volume competition between the water and oil phases suggests that balancing the proportions of these phases is very important for the construction of oil-water interfaces. The prepared PAA/PVP HGFMs exhibited considerable potential for use as lipase reactors, and the HGFMs exhibited good stability (in terms of the pH and temperature). The catalytic efficiency of HGFMs can be enhanced by infinite stacking in the longitudinal direction to form an enzyme membrane.

Although the method used to gel the PAA and PVP fibers was simple and efficient, the mechanical properties of the PAA/PVP HGFMs were inadequate. We hope to find more suitable gel fibers in the future. Overall, the current study provides a novel concept for efficient lipase catalysis and demonstrates the excellent potential of amphiphilic gel fibers for use as an enzymatic membrane reactor for lipase. We expect that this study will be a beneficial exploration for expanding the lipase catalytic system

## 4. Materials and Methods

### 4.1. Materials

Lipases from *Burkholderia cepacia* (BCL) and p-nitrophenyl phosphate (*p*-NPP) were purchased from Sigma–Aldrich (St. Louis, MI, USA). Polyacrylic acid (PAA) was purchased from Fujifilm Wako Pure Chemical Corporation (Chuo-Ku, Japan). Polyvinyl pyrrolidone (PVP) was purchased from Boai Nky Pharmaceuticals Ltd. (Jiaozuo, China). Polyamide 6 (PA6) was provided by DuPont Co., Ltd. (Wilmington, DE, USA). Butyl methacrylate (BMA) was purchased from Shanghai Aladdin Biochemical Technology Co., Ltd. (Shanghai, China). Polyacrylonitrile (PAN) was purchased from Hefei Sipin Technology Co., Ltd. (Hefei, China). Dodecane was purchased from Shanghai Macklin Biochemical Co., Ltd. (Shanghai, China). N, N-dimethylformamide (DMF) was purchased from Damao Chemical Reagent Factory (Tianjin, China). All other chemicals used in this study were at least analytical reagent grade.

### 4.2. Preparation of HFMs

Electrospinning Solution 1 was prepared by dissolving 8% (*w*/*w*) PAA and 0.1% sodium chloride (*w*/*w*) in an ethanol/water mixed solvent (19/1, *w*/*w*). The electrospinning parameters for PAA were as follows: the positive voltage was 16 kV, the negative voltage was −2 kV, and the electrospinning rate was 0.05 mm/min (1 mm/min corresponded to 0.1234 mL/min). Electrospinning Solution 2 was prepared by dissolving 10% (*w*/*w*) PVP in ethanol. The electrospinning parameters for PVP were as follows: the positive voltage was 12 kV, the negative voltage was −2 kV, the electrospinning distance was 14 cm and the electrospinning rate was 0.08 mm/min (1 mm/min corresponded to 0.1234 mL/min). The two polymer solutions were separately loaded into 5-mL syringes connected to an independent syringe pump. Two syringes were electrospun simultaneously for 8 h. During the electrospinning process, the humidity was controlled at 35 ± 5% and the temperature was 28 °C ± 3 °C.

### 4.3. Preparation of HGFMs

The PAA/PVP HFMs successfully converted into PAA/PVP HGFMs by a two-step crosslinking procedure: thermal polymerization followed by UV polymerization and then they were cut to the desired size (the weight of each HGFM was 4.5 ± 0.3 mg). The thermal polymerization process consisted of heating the PAA/PVP HFMs in an oven at 160 °C for 45 min. The UV polymerization process consisted of exposing the heat-treated PAA-PVP HFMs to UV light (400 W, 50 Hz) for 15 min.

### 4.4. Fourier Transform Infrared Spectroscopy

The changes in the chemical groups and structure of the PAA/PVP HFMs upon transformation to the PAA/PVP HGFMs were determined by Fourier transform infrared spectroscopy (Nicolet IS10, Thermo Nicolet Corporation, Madison, WI, USA).

### 4.5. Morphological Analysis of HFMs and HGFMs

The morphologies of the HFMs and HGFMs were imaged using scanning electron microscopy (SEM, Zeiss G300, Carl Zeiss AG, Oberkochen, Baden-Wurttemberg, Germany) and laser confocal scanning microscopy (CLSM, Olympus FV3000, Olympus Corporation, Tokyo, Japan). As preparation for CLSM observation of HFMs, Nile blue (0.1%, *w*/*w*) and Nile red (0.1%, *w*/*w*) were added to Electrospinning Solutions 1 and 2, respectively. In preparation for CLSM observation, the HGFM samples were soaked in a Nile blue solution (0.1%, *w*/*w*) for 12 h and then soaked in a Nile red solution (0.1%, *m*/*v*) for an additional 12 h. The HGFMs excitation wavelengths were 640 nm for Nile blue and 488 nm for Nile red.

### 4.6. Wettability of HGFMs

A contact angle analysis was performed to evaluate the wettability of the surface of the HGFMs. The water contact angle (WCA) and oil contact angle (OCA) of the HGFMs were measured by an optical contact angle meter (OCA, JC2000D5 M, China) using ultra-pure water and dodecane as wetting liquids, respectively.

### 4.7. Swelling Properties of HGFMs

The prepared HGFMs were cut into pieces with a radius of 0.6 cm, soaked in anhydrous ethanol for 12 h, dried in an oven until the quality remained unchanged, discharged into ultrapure water and soaked for 12 h, and dried in an oven until the quality remained unchanged; residual uncrosslinked fibers were then removed. The dried sample was soaked in ultra-pure water or dodecane. At a given time, the water swelling ratio (SR) and dodecane adsorption ratio (AR) of the HGFMs were calculated by Equations (1) and (2), respectively:

(1) $$\mathrm{SR}\;(\%)=\frac{{\;m}_{w}{\;-\;m}_{0}}{m_{0}\;}\times 100$$

(2) $$\mathrm{AR}\;(\%)=\frac{{\;m}_{d}{\;-\;m}_{0}}{m_{0}\;}\times 100$$

where $m_{0}$ represents the weight of the HGFM samples before soaking (g),${\;m}_{w}$ represents the weight of the swollen HGFM samples at the considered time (g) and ${\;m}_{d}$ represents the weight of the HGFM samples with adsorbed dodecane at the considered time (g).

The dried sample was soaked in ultra-pure water for 12 h to swell completely. The total swelling ratio (TSR) of the HGFMs at a given time was calculated by Equation (3):

(3) $$\mathrm{TSR}\;(\%)=\frac{{\;m}_{2}{\;-\;m}_{1}}{m_{1}\;}\times 100$$

where $m_{1}$ represents the weight of the HGFM sample after being soaked (g) and $m_{2}$ represents the weight of the HGFM sample swollen with dodecane at the considered time (g).

### 4.8. Lipase-Holding Capacity of HGFMs

The lipase dissolved in the PBS solution was adsorbed on the hydrogel fiber membrane, and the protein concentration of the enzyme solution after swelling of the HGFMs was measured using a standard BCA kit (Beijing Solarbio Science and Technology Co., Ltd., Beijing, China). The holding capacity (HC, mg/g) of the HGFMs for lipase at this time was determined by Equation (4):

(4) $$\mathrm{HC}=\frac{m_{4}-{\;C}_{t}\frac{m_{6}-{\;m}_{5}}{\rho }}{m_{5}}$$

where $m_{4}$ represents the quantity of lipase in the original lipase-PBS solution (g), C_t_ represents the concentration of lipase that determined by BCA kit (mg/mL), ρ represents the density of the lipase solution (4 mg/mL), and $m_{5}$ and $m_{6}\;$represent the weights of the HGFM sample before and after being soaked (g), respectively.

### 4.9. The Lipase Enzymatic Activity

The prepared HGFMs were cut into small discs with a radius of 0.6 cm, and the HGFMs discs were swelled by dropwise addition of 50 μL of lipase solution (4 mg/mL) for a swelling time of 6 h. Then, the swollen HGFMs discs were placed in 2 mL of a dodecane solution (4 mg/mL) containing p-nitrophenyl palmitate (*p*-NPP), and the catalytic reaction was allowed to proceed. After 30 min of reaction, the reaction was terminated by the addition of 2 mL of absolute ethanol, and the product of the catalytic reaction, p-nitrophenol (*p*-NP), was extracted using 2 mL of PBS solution (0.1 mol/L, pH = 7). The absorbance of the product was measured at a wavelength of 410 nm by a microplate reader (Tecan Infinite M200 Pro, Tecan, Männedorf, Switzerland). The content of *p*-NP (μmol) was converted by the absorbance. The total activity (U) of lipase was defined as the efficiency of the total content of *p*-NP produced per minute, and the specific enzyme activity of lipase (U/mg) was defined as the enzyme activity per unit of lipase loading capacity (mg). For the comparative analysis, the minimum specific activity (or total activity) of a sample in a group was defined as 100% of the relative specific activity (or total activity) and used to calculate the relative specific activity of the other samples in the group. The lipase activity was determined for the control (a macroscopic “oil-up/water-down” system with isooctane containing *p*-NPP as the oil phase and PBS containing lipase as the water phase), for which the cross-sectional area of the macroscopic “oil-up/water-down” system (the area of the oil–water interface in the horizontal direction) and the contents of lipase, the water phase, and the oil phase were completely the same as for the samples in the group.

### 4.10. Construction of the Enzyme Membrane Reactor

HGFMs discs with a radius of 0.6 cm were assembled using a simple string and a fine needle leaving a gap between discs and added to 2 mL of a dodecane solution containing *p*-NPP (4 mg/mL). The catalytic reaction was carried out for 30 min (15 min for special experiments). The contents of the water phase, oil phase and lipase in the “oil-up/water-down” system of the control group were the same as those for the HGFMs.

## Data Availability

The data used to support the findings of this study are available from the corresponding author upon request.

## Supplementary Materials

The following supporting information can be downloaded at: https://www.mdpi.com/article/10.3390/gels8120812/s1, Figure S1: FTIR spectroscopy of PAA fibers before and after thermal crosslinking (a) and PVP fibers before and after UV crosslinking (b); Figure S2: SEM images of PAA/PVP HFGMs under different thermal crosslinking times. Thermal cross-linking time: (a) 15 min, (b) 45 min, (c) 75 min. The electrospinning speed ratio of PAA /PVP was 5/8. The UV crosslinking was 15 min; Figure S3: SEM images of PAA/PVP HFMs and PAA/PVP HGFMs (a), PAA/PA6 HFMs and PAA/PA6 HGFMs (b), PAA/PAN HFMs and PAA/PAN HGFMs (c). The blue box represents PAA fibers and PAA gel fibers, the red box represents PVP fibers and PVP gel fibers, the orange box represents PV6 fibers and the green box represents PAN fibers.
