# Supplementary material for: Construction of a Novel Lipase Catalytic System Based on Hybrid Membranes with Interwoven Electrospun Polyacrylic Acid and Polyvinyl Pyrrolidone Gel Fibers
Source: Gels. 2022 Dec 10;8(12):812. doi: 10.3390/gels8120812 (PMC9777577; doi:10.3390/gels8120812)
Supplement: Supplementary file 1 [file gels-08-00812-s001.zip › gels-2079377-supplementary.pdf]

gels

MDPI

Supplementary Materials

Construction of a Novel Lipase Catalytic System Based on Hybrid Membranes with Interwoven Electrospun Polyacrylic Acid and Polyvinyl Pyrrolidone Gel Fibers

Ziheng Wang, Shumiao Lin, Qianqian Zhang, Jinlong Li * and Sheng Yin *

Methods

Preparation of HFMs:

PAN-electrospinning solution was prepared by dissolving 10% PAN (w/w) in ethanol/BMA (1:1). The electrospinning parameters for PAN were as follows: The positive voltage was 12 kV, the negative voltage was -2 kV, electrospinning distance was 12 cm and the electrospinning rate was 0.05 mm/min (1 mm/min represented 0.1234 mL/min). PA6-electrospinning solution was dissolving 15% PA6 (w/w) in formic acid/acetic acid mixed solvent (v/v: 1/1). The electrospinning parameters for PA6 were as follows: The positive voltage was 14 kV, the negative voltage was -2 kV, electrospinning distance was 13 cm and the electrospinning rate was 0.08 mm/min (1 mm/min represented 0.1234 mL/min). The PAA solution-electrospinning and PAN-electrospinning solution (or PA6-electrospinning solution) were separately loaded into 5 mL syringes connected to an independent syringe pump. Two syringes were electrospun at the same time, and the spinning time was 8 h. The humidity during electrospinning was controlled at 35±5%, and the temperature was 28 °C±3°C.

Preparation of HGFMs:

The PAA/PAN (or PAA/PA6) HFMs were cut to the desired size, then successfully converted into PAA/PAN (or PAA/PA6) HGFMs underwent a crosslinking procedure. In the thermal polymerization process, the PAA/PA6 HFMs were heated in an oven at 160 °C for 45min. The PAA/PAN HFMs were heated in an oven at 160 °C for 10min, the heated PAA/PAN HFMs were exposed to UV light (400W,50Hz) for 20min.

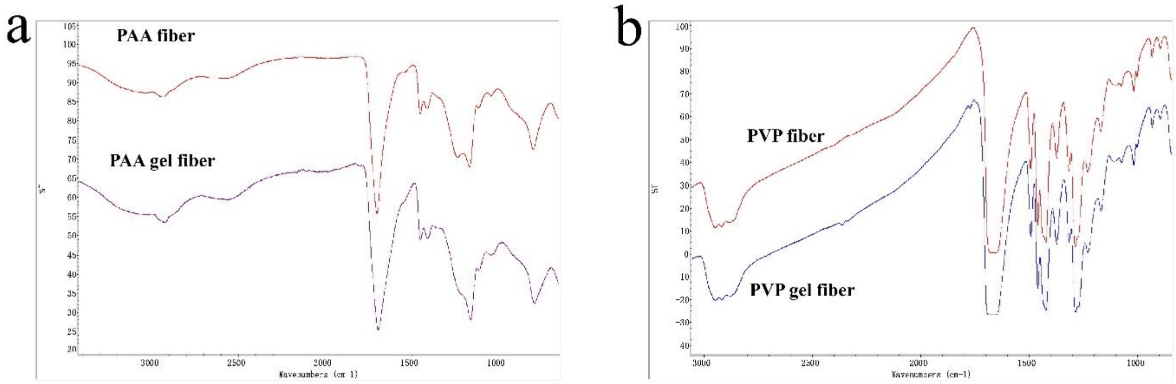

Figure S1. FTIR spectroscopy of PAA fibers before and after thermal crosslinking (**a**) and PVP fibers before and after UV crosslinking (**b**).

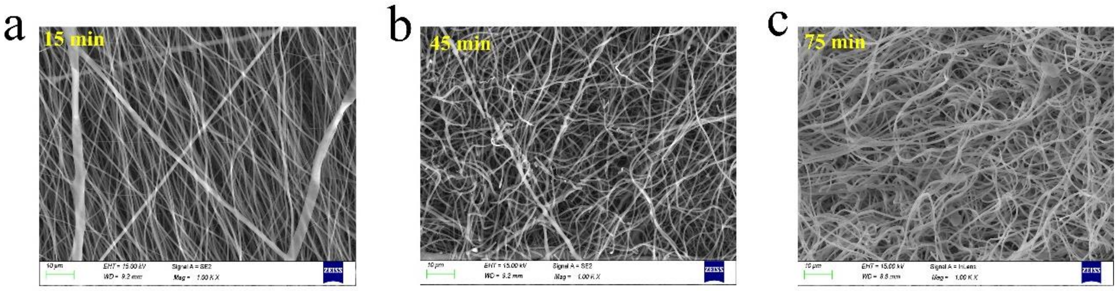

Figure S2. SEM images of PAA/PVP HFGMs under different thermal crosslinking times. Thermal crosslinking time:(**a**) 15min, (**b**) 45 min, (**c**) 75 min. The electrospinning speed ratio of PAA /PVP was 5/8. The UV crosslinking was 15 min.

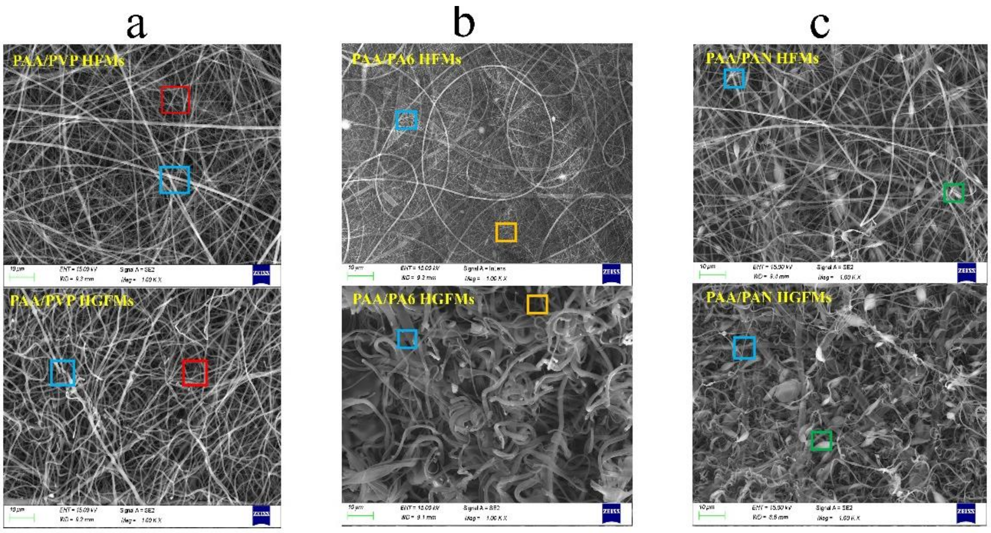

Figure S3. SEM images of PAA/PVP HFMs and PAA/PVP HGFMs (**a**), PAA/PA6 HFMs and PAA/PA6 HGFMs (**b**), PAA/PAN HFMs and PAA/PAN HGFMs (**c**). The blue box represents PAA fibers and PAA gel fibers, the red box represents PVP fibers and PVP gel fibers, the orange box represents PV6 fibers and the green box represents PAN fibers.
